# The Care of Peptic Ulcer Patients

**Published:** 1969-10

**Authors:** Norman C. Tanner


### THE CARE OF PEPTIC ULCER PATIENTS

**Norman C. Tanner**

In 1946 I was invited to speak to the Bristol Medico-Chirurgical Society on the management of peptic ulcer, and it is very appropriate that I should have this opportunity of reviewing the situation twenty three years later in 1969.

Let me first review the situation as it in fact exists today, which is identical with the position twenty-three years ago! The average patient, say a young man of between 20 and 30 years of age, begins to suffer from epigastric discomfort when he is hungry, and pain wakes him at nights. He consults his doctor who gives him alkalies and advice about diet. It is in the nature of ulcer disease, indeed its most typical feature, that the symptoms tend to come and go, remit and relapse periodically. A remission of symptoms as we know from gastroscopic observation does not mean that the ulcer is healed. Seeing a doctor or trying a new drug is often followed by a remission of symptoms. Thus the patient finds that the alkalies relieve his pain and a few days later he enters a remission, all his discomforts go, he can eat anything without pain and imagines he is cured. A few months later the episode recurs. This time it may last a little longer but with the help of his alkaline tablets he can continue with his work, but soon he has his next remission and again he finds he needs no more tablets and again is well. The third relapse of pain may well be treated again by advice on frequent small feeds and a new bottle of medicine, perhaps in addition a note to go to see a specialist. At the hospital he is X-rayed. Even if he has a duodenal ulcer there is in the earlier stages a 15% chance that no abnormality will be found in his duodenum or stomach. In this case he will be reassured, told about the effects of stress on his stomach and given a sedative. Or, if his X-ray shows a duodenal deformity he may on this occasion be given a printed diet sheet advising him to take small frequent meals of carbohydrate and fat. This diet, which is far more suitable for an infant during the process of weaning, can be guaranteed to change our hitherto anxious but lean young man into an anxious young man with a premature middle-age spread, with fatty infiltration in his myocardium and a reduced life expectation. He will again be given tablets to relieve pain. So things will go on, in about 90% of the ulcer cases I see, until our patient gets a haemorrhage, or until he gets so tired of the recurrent pain that he seeks surgical aid.

What is the *object* of medical treatment, is it merely to palliate or is it to cure the ulcer and keep it healed? Perhaps we should ask, what *can* we do medically? In my talk to the Bristol Medico-Chirurgical Society in 1946 I described my experiences with different forms of treatment of gastric ulcer, all studied under gastroscopic control (Tanner 1946).

| | |
|---|---|
| GASTRIC ULCERS treated first six months, 1943 ... | 108 |
| Number repeatedly examined in hospital to ascertain time required for healing reasonably accurately ... | 49 |
| Flat in Bed. 15 cases. | |
| Average age ... ... ... ... ... ... | 53 years |
| Average length of history ... ... ... ... | 8.6 years |
| Average time required for healing ... ... ... | 34 days |

Fowler position. 23 cases.

| | |
|---|---|
| Average age ... ... ... ... ... ... | 54 years |
| Average length of history ... ... ... ... | 7.4 years |
| Average time required for healing ... ... ... | 49 days |

Trendelenberg position. 11 cases.

| | |
|---|---|
| Average age ... ... ... ... ... ... | 55 years |
| Average length of history ... ... ... ... | 10 years |
| Average time required for healing ... ... ... | 35 days |

I then found that diet and alkali *and* bed rest led to ulcer healing in almost every case, but also discovered that when the drugs and the diet were eliminated, there was no reduction in the speed of healing. Diet and alkalies for the ambulant patient helped to allay his symptoms but played little or no part in producing ulcer healing, and I came to the conclusion that *recumbency* was the all important factor in leading to the cure of peptic ulcer.

Since that date Richard Doll and his associates have investigated the factors producing ulcer healing and came to a well nigh identical conclusion (Doll 1964), that bed rest is the only factor certain to promote ulcer healing.

Yet the majority of physicians still treat their patients with sloppy food and alkalies. Most American text books of medicine summarize medical treatment as giving alkalies in order to suppress pain and enable the patient to work. I was in Zurich last week and one of the U.S. trained gastro-enterologists said he never treated his uncomplicated ulcer patients with bed rest, they were kept comfortable and at work with small frequent meals and alkalies. Obviously if the patient needs alkalies to relieve pain he has an active ulcer, and so this is tantamount to agreeing to let the patient continue working despite having an active peptic ulcer. This is a policy of despair and merely keeps the patient going until he is ready for surgical treatment.

Surely we should set our sights higher than this! I would like to outline our own principles of ulcer care. First of all it should be regarded as a matter of urgency to heal an ulcer, and so we must use the method which acts most rapidly. Why is it a matter of urgency? With regard to gastric ulcer, it is urgent because we must make quite sure we are not dealing with an ulcer-cancer, and the best proof that an ulcer is benign is to see it heal. Secondly the more chronic and fibrotic an ulcer is allowed to become, the longer it will take to heal. A deep penetrating ulcer of only a few weeks history may heal in ten days, whereas the ulcer of years duration will require 35 to 45 days bed rest and the end result may be a severely deformed stomach or duodenum. In the 1940's I thought that the reason that ulcers relapse may be that we discharge the patient before the ulcer is completely healed. Thus in that period I made absolutely certain of ulcer healing, by gastroscopy, before discharging the patients. Some 5 to 12 years later Dr. Swynnerton followed up these cases, and to my disappointment we found that although this had been the group of ulcers considered most suitable for medical treatment, three quarters had relapsed.

**Gastric Ulcer 5-12 Years after Medical Treatment (178 Survivors Contacted)**

| | No. | % |
|---|---|---|
| Recurrence requiring gastrectomy ... | 57 | 32 |
| Frequent ulcer recurrences ... ... | 44 | 24.7 |
| Occasional recurrences ... ... | 35 | 19.7 |
| No recurrences ... ... ... | 42 | 23.6 |
| | 178 | |

Following that period, therefore, it became evident to me that we no longer need to look for a medical cure for ulcers; we have it in bed rest. What we do need is a medical method to prevent ulcer relapse.

Strict dieting is the favourite advice given. This is a soft diet, with a long list of things to avoid. What is the result of the strict diet?

1. It fattens the patient making him eventually a worse surgical risk.
2. The soft diet leaves the stomach more rapidly and so the ulcer scar remains longer in contact with unbuffered gastric juice.
3. Our conscientious patients, if they eat away from home, go through great anxieties before each such meal because of the difficulties of getting their ulcer diet in a restaurant, and this anxiety leads to great hypersecretion, so damaging the ulcer scar far more than taking the restaurant's *plat du jour* of roast beef and Yorkshire pudding would have done.
4. Most duodenal ulcer patients secrete excessive volumes of digestive juices and so they are *more* capable of digesting food than is the normal individual. We advise patients not to eat fried foods mainly because during ulcer relapse such foods cause more pain than soft foods. But if the patient has pain, then his ulcer has relapsed and he should be in bed, or be considered for surgery. If he has no pain and the ulcer is healed, then he can deal with fried foods as well as any other individual.

Therefore when the patient's ulcer is healed, I believe he will have less anxiety and he is less likely to relapse if he is merely instructed to eat at regular intervals but to take a normal diet, preferably without any excess of carbohydrate or of alcohol.

I think that everyday experience shows that strict dieting has failed and will *not* prevent ulcer relapse. We know that a certain level of acid secretion is necessary to cause relapse of an ulcer. Thus it is after the ulcer is healed and when the patient returns to the stresses and strains of everyday life that he needs drugs to reduce his acid secretion. Here we have made some advances since 1946. First of all we have inexpensive well tried non-absorbable antacids which can be given for years without risk of alkalosis, and which are nowadays dispensed in elegant and convenient tablet form, aluminium hydroxide, magnesium trisilicate, aluminium glycinate, etc. These are usually prescribed in too small a quantity to be effective, or worse, still used only for pain relief. I believe our now well healed and painfree patient should take an appropriate dose about ten times a day to protect his scar. Alternatively we have the anticholinergic drugs. Possibly the one with least side effects and most suitable for long term administration is poldine methylsulphate (Nacton) (Hunt 1966) which need be given only four times a day, on waking, midday, before the evening meal and perhaps most important, on retiring at night.

How long must our patient continue on this regime, bearing in mind that the risk of ulcer breakdown may be lifelong? I have known a nonogenerian whose duodenal ulcer perforated. I tell my patients that it is a life sentence of antacid therapy, and by this I mean quite as long as Her Majesty's judges give for murder. During holidays or especially anxiety-free periods, the drugs can be relaxed, but they should be resumed particularly during times of worry or stress. If the causation of the ulcer is related to special stress then the patient should continue the anticholinergic until this period is over.

What of carbenoxolone, liquorice? This is not suitable for prophylactic long-term administration because of its side effects, and in bed rest we have a more rapid method of producing ulcer healing.

### SURGERY

One of the reasons that so few patients have bed rest for their ulcer is the often expressed feeling of these patients that bed rest is a waste of their time. If in the out-patients department I advise patients to have two to three weeks bed rest and then be re-examined to confirm healing of the ulcer, they will often resist strongly. Yet, curiously enough, if I suggest coming into hospital for an operation, with 14 days in the hospital and six weeks convalescence before resuming work, they are very willing to co-operate. Therefore we have to explain that bed rest is positive therapy, and is their most rapid route to recovery and resumption of work with an intact stomach.

However, by the time we see them many patients have so much duodenal or gastric deformity from ulceration that the likelihood of the ulcer remaining healed is minimal, and so surgical management is preferable.

I am fortunate in that a number of physicians, mainly under the guidance of Dr. B. F. Swynnerton, have made careful studies of our ulcer patients five and ten or more years after operation.

Very briefly the Visick classification of our cases of partial gastrectomy for duodenal ulcer is as follows:—

**Classification of 100 Survivors of Gastrectomy for Duodenal Ulcer**

| | | | |
|---|---|---|---|
| Visick | I | 84 | 95% satisfactory (I–IIIS) |
| | II | 6 | |
| | IIIS | 5 | |
| | IIIU | 3 | |
| | IV | 2 | |

For total vagotomy with either pyloroplasty or with anterior juxta-pyloric gastro-jejunostomy, it is as follows:—

**Classification of Survivors of Vagotomy at 5 years-10 years**

| | with pyloroplasty | | with ant. juxtapyloric gastro-jejunostomy | |
|---|---|---|---|---|
| Visick I | 76 (38%) | 85.5% satisfactory | 46 (39%) | 87% satisfactory |
| II | 46 (23%) | | 19 (16%) | |
| IIIS | 50 (24.5%) | | 37 (32%) | |
| IIIU | 11 5.5% | | 7 6% | |
| IV | 18 9% | | 8 7% | |

The late, 11 to 21 years, follow-up of truncal vagotomy is on the whole very satisfactory, with normal serum protein, calcium, folate, and B12 values. The only biochemical disturbance was an occasional iron deficiency among the cases of vagotomy with gastro-jejunostomy, readily corrected by iron therapy.

**Vagotomy and Pyloroplasty**

Results 11-21 years later

| | | |
|---|---|---|
| Visick I | 45.5% | 97% |
| II | 42.5% | |
| IIIS | 9% | |
| IIIU | 3% | |
| IV | 0 | |

**Vagotomy plus Gastro-jejunostomy**

Results 11-21 years later

| | | |
|---|---|---|
| Visick I | 47.8% | 91.2% |
| II | 34.7% | |
| IIIS | 8.7% | |
| IIIU | 8.7% | |
| IV | 0 | |

11-21 years after 192 Truncal vagotomies

**Episodic Diarrhoea**

| | | Men | Women |
|---|---|---|---|
| Vagotomy and pyloroplasty ... | mild | 22.5% | 21% |
| | serious | 1.6% | 5% |
| Vagotomy and gastro-jejunostomy ... | mild | 29% | 27% |
| | serious | 0% | 0% |

11-21 years after 192 Truncal vagotomies

**Blood**

Average Haemoglobin

| | Men |
|---|---|
| after vagotomy + pyloroplasty ... ... | 14.3Gm% |
| after vagotomy + gastro-jejunstomy ... | 13.1Gm% |

Iron deficiency type.

It appears that on the whole there are only slight differences between the conditions of these patients after gastrectomy or after vagotomy, but after a consideration of the side effects of the various operations, particularly diarrhoea and dumping, the partial gastrectomy results for duodenal ulcer are minimally better. However vagotomy has the great advantage of an extremely small operative mortality, it should be well under 0.5%. For this very cogent reason therefore we must support it as the operation of choice for duodenal ulcer.

I have little doubt that in our next follow-up the results of vagotomy will be even better than those I have recorded, because of the present use of gastric or selective vagotomy. Preserving the nerve supply to the pancreas and small intestine undoubtedly reduces the incidence of episodic diarrhoea. Now we are left with the dumping symptoms, fullness and hypoglycaemia-like attacks as the main problem. These are probably the result of the gastric drainage rather than the vagal section, and so the next question to which we have to find an answer is, can we dispense with any form of drainage for pure 'gastric vagotomy' in cases with non-obstructed duodenal ulcer?

If we find that we can, we shall be well on the way to the ideal surgical solution of the duodenal ulcer problem.

### BIBLIOGRAPHY

Doll, R. (1964) Scot. Med. J., *9*, 183.

Hunt, J. N. (1966) "Progress in patients with peptic ulceration treated for more than 5 years with Poldine including a double blind study". Brit. Med. J., *2*, 13-16.

Tanner, N. C. (1946) "The Management of Peptic Ulcer". Bristol Med. Chir. J., *63*, 16-30.

